# Severe pressure ulcer caused by an electrode belt for monitoring electrical impedance tomography in two patients in the prone position

**DOI:** 10.1186/s40981-023-00675-z

**Published:** 2023-11-25

**Authors:** Takayuki Hasegawa, Keisuke Yoshida, Takahiro Hakozaki, Satoki Inoue

**Affiliations:** Division of Anesthesia and Pain Medicine, Fukushima Medical University Hospital, 1 Hikarigaoka, Fukushima City, Fukushima 960-1295 Japan

To the Editor,

Electrical impedance tomography (EIT) has emerged as a real-time dynamic monitoring technique for assessing pulmonary ventilation, which can be used to optimize mechanical ventilation for acute respiratory distress syndrome (ARDS) [1]. Also, prone ventilation is often performed for severe ARDS [2, 3]. Several studies have monitored the respiratory system using EIT during prone positioning [4, 5], and we believe that the combination of EIT monitoring and prone therapy will become more common in clinical practice for ARDS patients. Herein, we report two cases of pressure ulcers caused by the electrode belt of an EIT device (Enlight™ 2100, Medtronic, Minneapolis, MN, USA) placed on the chest during prone ventilation for ARDS in our intensive care unit. To the best of our knowledge, this is the first report of severe ulcers caused by an EIT device.

Case 1: An 81-year-old man (height 162 cm, body weight 62 kg, body mass index 23.6 kg/m^2^) who underwent subtotal esophagectomy for esophageal cancer developed pneumonia and severe ARDS on the third postoperative day. Lung-protective ventilation with neuromuscular blocking agents and EIT monitoring were performed with the patient in the prone position for 20 h. After prone ventilation, pressure ulcers were found in the left anterior axilla where the EIT electrode belt had been placed (Fig. 1a). After 3 months, the ulcers had not healed; rather, they had increased in size (Fig. 1b).

**Fig. 1:**
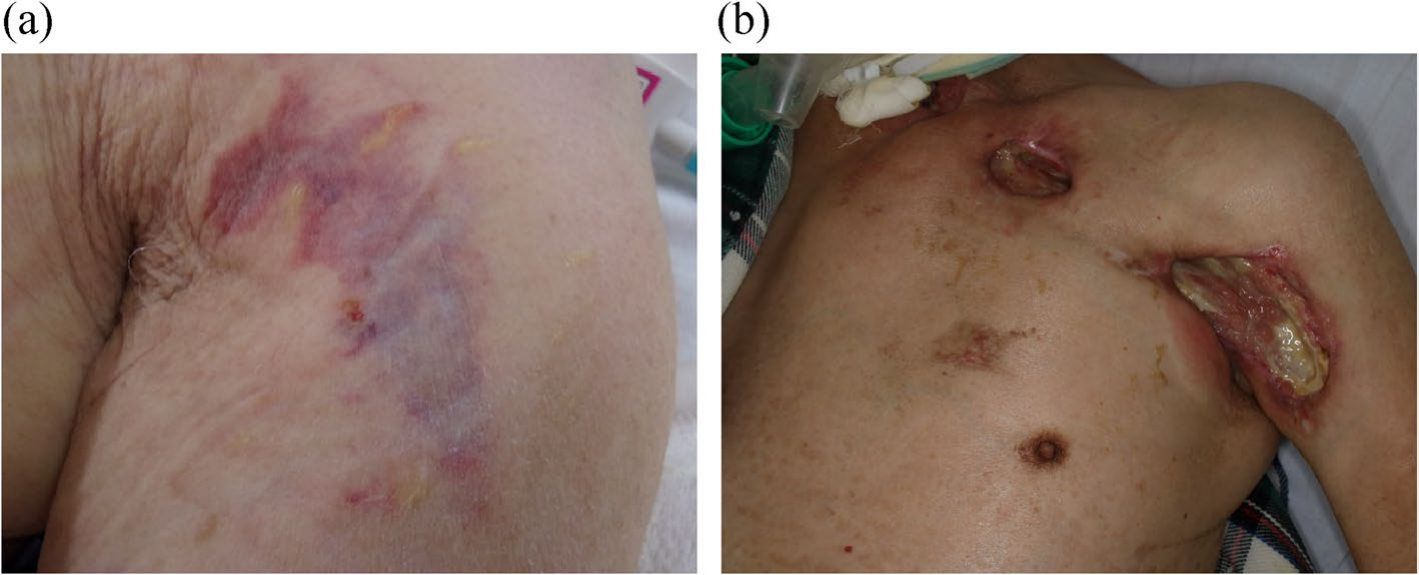
Skin lesions by an electrode belt for monitoring electrical impedance tomography in case 1. **a** Erythema and purpura in the left axilla and the upper arm immediately after prone to supine positioning. **b** Severe ulcer with extensive skin defects in the chest wall and the axilla 3 months later

Case 2: A 66-year-old man (height 170 cm, body weight 42 kg, body mass index 14.6 kg/m^2^) receiving chemotherapy for esophageal cancer developed bacterial pneumonia and severe ARDS. We used lung-protective ventilation with tracheal intubation and neuromuscular bloking agents, along with EIT monitoring, in the prone position for 19 h. After the prone ventilation, pressure ulcers were found on the anterior chest (Fig. 2).

**Fig. 2:**
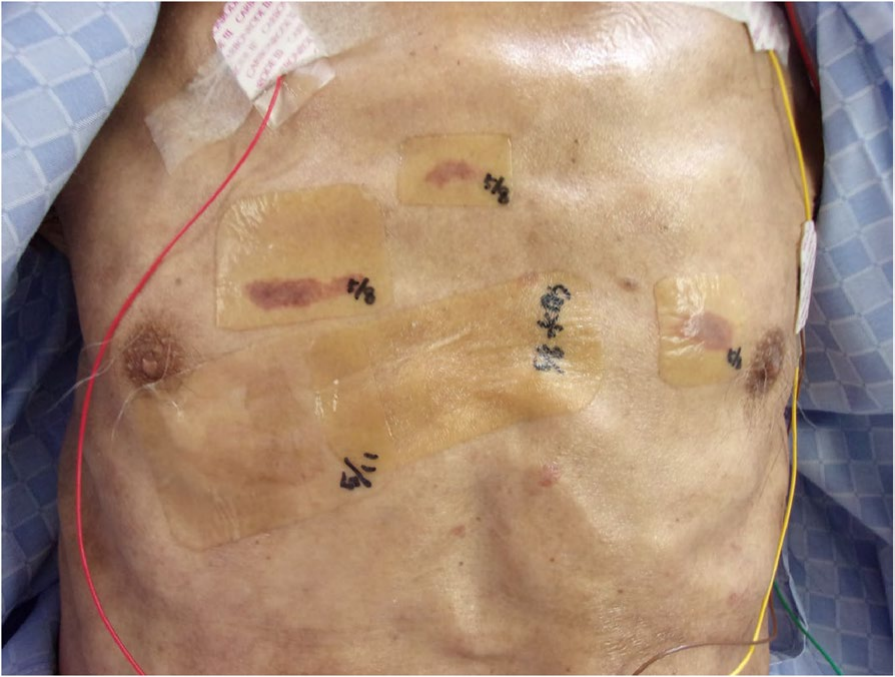
Skin lesions without defects in the anterior chest wall by an electrode belt for electrical impedance tomography immediately after prone to supine positioning in case 2

We propose that EIT may be a risk factor for pressure ulcers. These two patients developed their pressure ulcers on the sites where the EIT electrode belt was in contact with their skin. During prone ventilation, we used cushions and pillows to relieve pressure and performed decompression every 2 h to prevent pressure-induced skin damage. However, it is not easy to achieve fully effective decompression with the patient in the prone position. In addition, both patients had risk factors for pressure ulcers, such as male gender, age ≥ 60 years, and low body mass index [6]. The findings of these cases suggest that the use of EIT devices as well as the said risk factors may induce pressure ulcers, potentially leading to prolonged hospital stay; therefore, careful attention should be paid to pressure ulcers, especially when EIT is used in the prone ventilation.

## Data Availability

Not applicable.

## Abbreviations

EIT: Electrical impedance tomography
ARDS: Acute respiratory distress syndrome
